# Tetrodotoxin Poisoning Due to Pufferfish and Gastropods, and Their Intoxication Mechanism

**DOI:** 10.5402/2011/276939

**Published:** 2011-11-30

**Authors:** Tamao Noguchi, Kazue Onuki, Osamu Arakawa

**Affiliations:** ^1^Faculty of Healthcare, Tokyo Healthcare University, Setagaya, Tokyo 154-8568, Japan; ^2^Graduate School of Fisheries Science and Environmental Studies, Nagasaki University, Nagasaki 852-8521, Japan

## Abstract

Marine pufferfish generally contain a large amount of tetrodotoxin (TTX) in their skin and viscera, and have caused many incidences of food poisoning, especially in Japan. Edible species and body tissues of pufferfish, as well as their allowable fishing areas, are therefore clearly stipulated in Japan, but still 2 to 3 people die every year due to pufferfish poisoning. TTX is originally produced by marine bacteria, and pufferfish are intoxicated through the food chain that starts with the bacteria. Pufferfish become nontoxic when fed TTX-free diets in a closed environment in which there is no possible invasion of TTX-bearing organisms. On the other hand, TTX poisoning due to marine snails has recently spread through Japan, China, Taiwan, and Europe. In addition, TTX poisoning of dogs due to the ingestion of sea slugs was recently reported in New Zealand. TTX in these gastropods also seems to be exogenous; carnivorous large snails are intoxicated by eating toxic starfish, and necrophagous small-to-medium snails, the viscera of dead pufferfish after spawning. Close attention must be paid to the geographic expansion and/or diversification of TTX-bearing organisms, and to the sudden occurrence of other forms of TTX poisoning due to their ingestion.

## 1. Introduction

**Figure 1:**
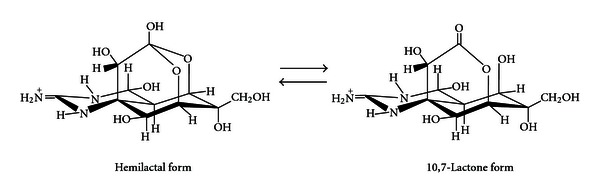
Chemical structure of TTX.

In Japan, tetrodotoxin (TTX) is the most common natural marine toxin to cause food poisoning, and it poses a serious hazard to public health. This toxin (C_11_H_17_N_3_O_8_; Figure 1) is a potent neurotoxin with a molecular weight of 319, whose various derivatives have been separated from pufferfish, newts, frogs, and other TTX-bearing organisms [1]. When ingested by humans, TTX acts to block the sodium channels in the nerve cells and skeletal muscles [2], and to thereby block excitatory conduction, resulting in the occurrence of typical symptoms and signs (Table 1) and even death in severe cases [3]. The lethal potency is 5000 to 6000 MU/mg (1 MU (mouse unit) is defined as the amount of toxin required to kill a 20-gram male mouse within 30 min after intraperitoneal administration), and the minimum lethal dose for humans is estimated to be approximately 10,000 MU (*≈*2 mg).

**Figure 2:**
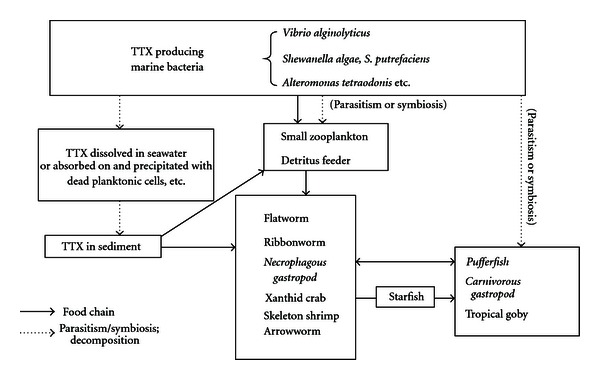
Proposed mechanism of TTX intoxication in marine animals.

 Since 1964 [4], the distribution of TTX has spread to animals other than pufferfish, including newts, gobies, frogs, octopuses, gastropods, starfish, crabs, flatworms, and ribbon worms (Table 2) [5, 6]. Pufferfish are thought to accumulate TTX through several steps of the food chain, starting from TTX production by marine bacteria (Figure 2) [6, 7]. TTX poisoning due to marine gastropods occurs not only in Japan [5], but also in China [3], Taiwan [3], Europe [8], and New Zeeland [9], suggesting further diversification of TTX-bearing organisms and therefore geographic expansion of TTX poisoning. In the present paper, we review TTX poisoning cases due to the ingestion of pufferfish and gastropods, and discuss the TTX intoxication mechanism of these organisms in an effort to contribute to the development of an effective means of protecting humans against TTX poisoning.

## 2. TTX Poisoning due to Pufferfish

Marine pufferfish of the family Tetraodontidae generally contain a large amount of TTX in their skin and viscera, especially the liver and ovary [6]. Accordingly, edible species and their body tissues, and the allowable pufferfish fishing areas have been clearly stipulated in Japan since 1983, but still several tens of people are poisoned by pufferfish annually, and 2 to 3 people die as a result of pufferfish poisoning (Table 3). The incidence in specializing restaurants is rare, and most cases of poisoning result when people with little knowledge of pufferfish toxicity cook a pufferfish that they caught or received from someone else and mistakenly eating strongly toxic parts such as liver and ovary. Some pufferfish fans dare to ingest the liver, believing that the toxin can be eliminated by their own special detoxification methods.

In October 2008, a 69-year-old male died at a hospital in Isahaya, Nagasaki Prefecture [10]. He stated that he cooked a “usubahagi” (a sort of thread-sail filefish “kawahagi”) that he caught by himself and ate its raw meat (sashimi) after dipping in a mixture of the liver and soy sauce. Approximately 30 minutes after ingestion, he felt numbness in his limbs, and 30 minutes later, he vomited and became comatose before being transported by an ambulance to the hospital. The doctor confirmed his death approximately 4 hours after ingestion, with an initial diagnosis of “ciguatera due to the ingestion of “kawahagi” liver, the possibility of TTX is not denied”. Thereafter, it was determined that the patient cooked a “kinfugu” (local name of pufferfish) with the “usubahagi,” but the liver was missing among the leftovers. We investigated the leftovers, and revealed that the “usubahagi” was nontoxic, but the “kinfugu” was actually a highly toxic species, “komonfugu” *Takifugu poecilonotus*, and 600 MU/g of TTX was detected in the skin. Furthermore, 0.7 MU/mL, 2 MU/mL, and 45 MU/g of TTX was detected in the blood, urine, and vomit of the patient, respectively, leading to the conclusion that this was a case of TTX intoxication due to the mistaken ingestion of *T. poecilonotus *liver.

Recently, the nonedible pufferfish *Lagocephalus lunaris*, which usually inhabits tropical or subtropical waters, has been frequently mixed up with edible species in Japanese coastal waters, posing a serious food hygiene problem. This pufferfish, which bears a very similar appearance to the almost nontoxic species *L. wheeleri*, also possesses high levels of TTX in their muscles [6, 11], caused 5 poisoning incidents in 11 patients due to mistaken ingestion in Kyushu and Shikoku Islands during 2008-2009. Though not as frequent as in Japan, many food poisoning cases due to ingestion of wild pufferfish have also occurred in China and Taiwan [3, 6].

## 3. TTX Poisoning due to Gastropods

**Figure 3:**
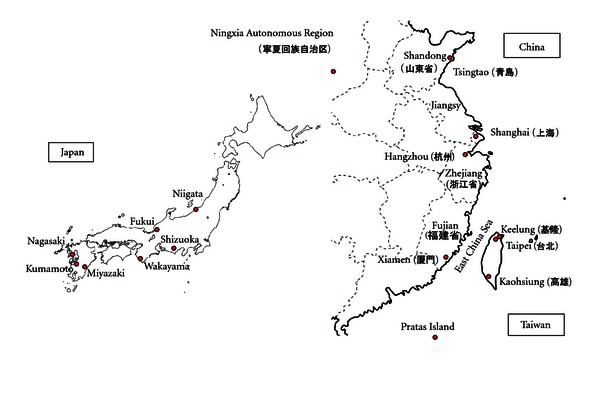
Maps showing the locations where TTX poisoning due to marine snails occurred in Japan, China, and Taiwan.

TTX-bearing gastropods and the food poisoning incidents due to their ingestion are summarized in Tables 4 and 5, and Figure 3.

### 3.1. Large Marine Snails

 Although the trumpet shell *Charonia sauliae *is not usually sold on the market, it is sometimes eaten locally in Japan. In December 1979, a man in Shimizu, Shizuoka Prefecture, Japan, ingested the digestive gland of *C. sauliae* and was seriously poisoned. He showed paralysis of his lips and mouth, and respiration failure, which are the typical symptoms and signs of pufferfish poisoning. TTX was detected for the first time in a marine snail, that is, the leftovers of *C. sauliae*, and the causative agent was therefore concluded to be TTX [12]. Similar poisonings occurred in 1 patient in the Wakayama Prefecture in December 1982, and in 2 patients in the Miyazaki Prefecture in January 1987.

 In *C. sauliae*, TTX localizes in the digestive gland, and other organs, including the muscle, are nearly nontoxic [12]. The digestive gland toxicity of *C. sauliae* collected from Shimizu Bay in 1981 ranged from 77 to 350 MU/g. A subsequent toxicity survey based on a total of 1406 digestive glands of *C. sauliae* from 7 prefectures indicated that the frequency of toxic specimens in each prefecture ranged from 19% to 87%. TTX or its derivative been also detected in closely related species, such as the frog shell *Tutufa lissostoma* [13] and the European trumpet shell *Charonia lampas lampus* [8], the latter of which caused TTX poisoning in Spain in 2007.

### 3.2. Medium Marine Snails

 The ivory shell* Babylonia japonica* is usually ingested as a side dish with sake. In June 1957, 5 persons were poisoned due to ingestion of the shellfish in Teradomari, Niigata Prefecture, and 3 of them died [14]. The causative substance was estimated to be TTX based on the facts that the symptoms and signs of the victims were similar to those of the pufferfish poisoning, and that TTX was later detected in *B. Japonica* collected from Kawajiri Bay, Fukui Prefecture in May 1980 [15].

In April 2004, a food poisoning incident resulting from the ingestion of the necrophagous marine snail *Nassarius (Alectricon) glans* occurred in Tungsa Island located in the South China Sea, Taiwan. Five patients were involved, and there were 2 deaths. The causative agent was identified as TTX by instrumental analyses [16, 17]. In a toxicity survey of 20 *N. glans* specimens collected from the same sea area, high toxicity was observed not only in the digestive gland, but also in the muscle (average of 538 and 1167 MU/g, resp.).

 TTX poisonings due to *N. glans *have also occurred in Japan recently [10]. In July 2007 in Nagasaki, Nagasaki Prefecture, a 60-year-old female developed a feverish feeling in the limbs, abdominal pain, and an active flush and edema in the face 15 minutes after ingesting the shellfish and was administered intravenous fluids at a clinic near her home. Thereafter, her condition worsened, and she developed dyspnea, whole-body paralysis, and mydriasis; she was finally transported to an emergency hospital. The patient required an artificial respirator for the first 3 days, but recovered enough to take breakfast on the 4th day. She unexpectedly relapsed after lunch, however, and developed respiratory arrest and was placed on the respirator again. She gradually recovered and was discharged from the hospital 3 weeks later.

Immediately after the incident, we investigated the leftover gastropods and detected a maximum of 4290 MU/g of TTX in the cooked muscles and digestive glands of *N. glans*. Moreover, during subsequent investigations, an extremely high concentration of TTX and a putative derivative of TTX, that is, a maximum of 10,200 MU/g (15,100 MU/individual) in the viscera and 2370 MU/g (9860 MU/individual) in the muscle, were detected in *N. glans* specimens collected from the same sea area as the ingested snail [18]. In this case, the once-recovered symptoms recurred after the patient began eating again. Although the reason is not clear, the recurrence might have been due to the digestion of a highly toxic, previously undigested tissue fragment of *N. glans* and absorption due to the resumption of meals, again exposing her respiratory center to a high concentration of TTX. In July 2008, another poisoning incident due to *N. glans* occurred in Amakusa, Kumamoto Prefecture.

### 3.3. Small Marine Snails

In association with the occurrence of TTX poisoning by *C. sauriae* in Shizuoka Prefecture in 1979, TTX screening was performed in several species of small marine snails in Japan. *Zeuxis siquijorensis* [19] and *Niotha clathrata* [20] were found to possess TTX or a TTX-like substance. There have been, however, no poisoning cases in Japan, as Japanese people do not typically feed on these species. On the other hand, inhabitants along the coast of the East China Sea in China and Taiwan have a long history of eating small marine snails, and *Zeuxis* spp. *N. clathrata,* and *Natica* spp. are generally sold at the supermarket or fish markets in these areas. From 1977 to 2004, more than 419 people were poisoned by ingesting these snails, and over 19 people died in Zhoushan, Fujian, and the Ninxia Hui Automous Region in China [3, 21–23]. Furthermore, poisoning cases have spread along the coasts from Fujian to Tsuingtao. In 1994 and 2001, similar poisonings occurred in the southern and northern parts of Taiwan, respectively, and the main causative substance was identified as TTX [23–25].

### 3.4. Sea Slugs

 From July to November 2009, 15 dogs were suddenly poisoned at the beaches adjacent to Hauraki Gulf, Auckland, New Zealand, all exhibiting similar symptoms, and 5 of them died. McNabb et al. [9] detected a very high level of TTX in the grey side-gilled sea slug *Pleurobranchaea maculate* found in tide pools near the beach and claimed that the dogs were poisoned with TTX by contact with the sea slugs. TTX was found in the eggs and larvae and distributed over the whole body with increasing concentrations toward the outer tissues in the adult sea slugs.

## 4. TTX Intoxication Mechanism of Pufferfish

Marked individual and regional variations are observed in pufferfish toxicity. In addition, the facts that the TTX of *C. sauliae* and *B. Japonica* comes from the food chain as described below and that several shell fragments of *Z. siquijorensis* are detected in the digestive tract of the toxic pufferfish *Takifugu pardalis* suggest that TTX contained by pufferfish is exogenous via the food chain [6, 7]. Moreover, many studies of TTX have revealed that (1) TTX is distributed over various organisms other than pufferfish, (2) marine bacteria primarily produce TTX (Table 6), (3) pufferfish become nontoxic when they are fed TTX-free diets in a closed environment in which there has been no invasion of TTX-bearing organisms, (4) such nontoxic pufferfish efficiently accumulate TTX when TTX is orally administered, and (5) pufferfish are equipped with high resistance to TTX, supporting the exogenous intoxication theory—a hypothesis that TTX is originally produced by marine bacteria, and pufferfish accumulate TTX through the food chain that starts with the bacteria [6, 7].

 To test (3), we investigated the toxicity of more than 8700 individual pufferfish that had been reared in an environment in which the invasion of TTX-bearers was prevented and were provided nontoxic diets in netcages in the sea, or in tanks with an open or closed circulation system on land, and confirmed that all the livers remained nontoxic (Table 7) [6, 25]. Production of nontoxic pufferfish can reduce the risk of food poisoning from eating toxic pufferfish and reduce the mortality rate. Moreover, this method might also contribute to maintain the Japanese food culture by reviving pufferfish liver dishes as a safe traditional food, which, although eaten previously, has been prohibited as a food since the regulation of 1983 in Japan. The transfer, accumulation, and elimination mechanisms of TTX taken up into the pufferfish body via food organisms remain unclear. We recently found that TTX administered intramuscularly to nontoxic cultured specimens of the pufferfish *Takifugu rubripes* was transferred first to the liver and then to the skin via the blood [26]. Matsumoto/Nagashima et al. demonstrated that, unlike general nontoxic fish, the liver tissue of *T. rubripes* is equipped with a specific TTX-uptake mechanism [27–29], and using a pharmacokinetic model showed that TTX introduced into the pufferfish body is rapidly taken up into the liver via the blood [30, 31]. These findings indicate that marine pufferfish are endowed with a mechanism by which they transport TTX specifically and actively. TTX-binding proteins have been isolated from the blood plasma of marine pufferfish, and may be involved in the transportation mechanism [32, 33]. 

 In wild pufferfish, the liver and ovary usually have strong toxicity, whereas the muscle and testis are weakly toxic or nontoxic [6]. In addition, the toxicity varies with the season, usually reaching the highest level during the spawning season (March to June in Japan), indicating sexual differences in pufferfish toxicity and that maturation may affect toxin kinetics in the pufferfish body. Recently, we investigated seasonal changes in tissue toxicity and the amount and forms of TTX in the blood plasma using wild specimens of the pufferfish *T. poecilonotus* and demonstrated that maturation greatly affects the intertissue transfer and/or accumulation of TTX via the bloodstream [34].

## 5. TTX Intoxication Mechanism of Gastropods

### 5.1. Large Marine Snails

 The trumpet shell *C. sauliae *is a carnivorous marine snail, and fragments of the starfish *Astropecten polyacanthus* were detected in the digestive tract of the specimens collected from Shimizu Bay in association with the food poisoning in 1979. The starfish were toxic, and the toxic molecule was identified as TTX [35]. The closely related species *A. scoparius* [36] and *A. latespinosus* [37] also had TTX. Moreover, an experiment in which nontoxic *C. sauliae* were fed toxic starfish demonstrated that the TTX of *C. sauliae* is derived from these starfish, namely, their food source [35, 38]. The starfish of genus *Astropecten* are also carnivorous, and their toxin is also estimated to come from their food.

### 5.2. Medium Marine Snails

 The ivory shell* B. japonica* is necrophagous and feeds on the muscles and viscera of dead fish. In the Hokuriku and Joetsu districts along the Japan Sea where Sakajiri Bay is located, and TTX intoxication of *B. japonica* was recognized in 1980 [15], fishermen are familiar with the feeding habits of *B. japonica* and catch them using the viscera of dead toxic pufferfish *Takifugu niphobles* as bait. We performed a similar experiment with *C. sauliae* and observed that *B. japonica *preferentially ate dead pufferfish viscera, thereby accumulating TTX. It is presumed that the *B. japonica* that caused poisoning in Teradomari of the Joetsu district were intoxicated with TTX by a similar mechanism.

**Figure 4:**
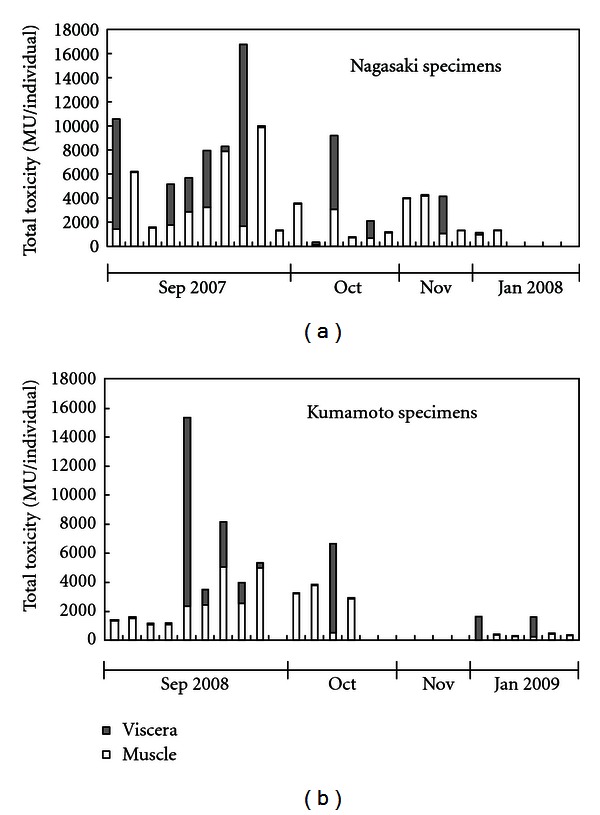
Toxicity of *N. glans* collected from Tachibana Bay, Nagasaki Prefectuire (a) and Miyanokawachi Bay, Kumamoto Prefecture (b).

 Although the TTX intoxication mechanisms of *N. glans* in Tsungsa Island as well as Nagasaki and Kumamoto Prefectures are unclear, the necrophagous characteristics of the snail suggest that dead pufferfish viscera are one of the origins of TTX. The toxicity of the Nagasaki/Kumamoto specimens of *N. glans* collected from September to January was highest in September, and gradually decreased thereafter (Figure 4) [10, 18]. There are no data on the other months, but both poisoning incidents in Nagasaki and Kumamoto occurred in July, indicating that the *N. glans* had already accumulated a high concentration of TTX that month. In Japan, *T. niphobles* comes *en masse* to the seashore to spawn their eggs in June, and die shortly thereafter. The spawning season of *T. niphobles* almost corresponds to the intoxication season of *N. glans*, indicating a possibility that *N. glans* is intoxicated by feeding on the mass of dead *T. niphobles* at the sea bottom. 

### 5.3. Small Marine Snails

The occurrence of food poisoning cases in China and Taiwan is concentrated from spring to early summer (Table 5), somewhat earlier than that of the Nagasaki/Kumamoto incidents. On the other hand, the season during which toxic pufferfish approach the seacoast in a group to spawn is earlier in China and Taiwan than in Japan, as the latitude of the area where the poisonings occur is lower than that of Japan proper (Figure 3). Therefore, the season when poisonings occur appears to correspond to the spawning season of toxic pufferfish. The small marine snails that have caused food poisonings in China and Taiwan are all necrophagous, having the same feeding habit as *B. japonica* and *N. glans*, and seem to be intoxicated by the same mechanism; they accumulate TTX by feeding on the viscera of toxic pufferfish that died after spawning.

 In this context, TTX has been found to act as an attractant to toxic marine snails. In our experiment using 8 toxic and 2 nontoxic snail species to investigate the attracting effect of TTX, we observed a significantly positive correlation between toxicity and comparative attracting variations in toxic species, whereas nontoxic species showed a negative response to TTX [39]. Carnivorous or necrophagous marine snails generally live at the sea bottom, and their habitat, including their prey and food sources, is very limited. Under such conditions, the snails may be endowed with the ability to detect TTX-bearing foods and to ingest them selectively as a species-specific characteristic.

 Although necrophagous small snails ingest TTX-containing foods selectively, they also have access to a diet contaminated with paralytic shellfish poison (PSP; i.e., a group of neurotoxins produced by certain species of dinoflagellates, and the main component, STX, has an almost equivalent molecular size and action mechanism to TTX [40]). In such cases, they accumulate not only TTX but also PSP, as seen in* Natica lineate* [41], *Niotha clathrata* [23, 24], and *Zeuxis scalaris* [23, 24] in Pingtung, Taiwan. This is also the case in the toxic crabs *Zosimus aeneus* in the Philippines [42] and Taiwan [43], and *Atergatis floridus* in Taiwan [44].

### 5.4. Sea Slugs

 According to McNabb et al., sea slugs are carnivorous scavengers living in the shallow subtidal crustose turf/benthic algal communities [9]. The mechanisms of their TTX intoxication remain uncertain. Sea slugs are generally not used for human food, but the dog poisonings may be viewed as a warning to human public hygiene. Namely, if their intoxication is caused by a route other than the presently known food chain, this may suggest a novel original organism of TTX, and the food chain that begins with this organism may contaminate seafood previously thought to be safe with TTX.

## 6. Conclusion

TTX was originally named after the family name, Tetraodontidae, of pufferfish as their exclusive toxin, and TTX poisoning due to ingestion of pufferfish has long been recognized. TTX poisoning due to gastropods, however, has also begun to occur frequently, posing a serious food hygiene problem. TTX is exogenous to both pufferfish and gastropods, and they are thought to ingest it from toxic food organisms and to accumulate the TTX in specific organs. Interestingly, it is presumed that live pufferfish ingest/accumulate TTX from necrophagous small or medium marine snails, while on the other hand, these snails ingest/accumulate the toxin from dead pufferfish. Thus, it is possible that the TTX produced by bacteria not only transfers to higher organisms through the food chain, but that it also partly circulates between certain organisms (Figure 2).

As described above, the pufferfish *L. lunaris,* originally inhabiting tropical to subtropical sea areas, now frequently appear in the temperate coastal waters of Japan, and dog poisonings due to sea slugs have suddenly begun to occur in the Southern Hemisphere. Such facts indicate the possibility of further geographic expansion and/or diversification of TTX-bearing organisms, or of TTX contamination of seafood caused by a change in the marine environment, such as an increase in the water temperature due to global warming. Careful attention must be paid to this point from the food hygiene perspective for the future.

## Figures and Tables

**Table 1:** Symptoms of TTX poisoning.

Degree	Characteristic symptoms
First	Neuromuscular symptoms (paresthesia of lips, tongue, and pharynx; taste disturbance; dizziness; headache; diaphoresis; pupillary constriction); gastrointestinal symptoms (salivation, hypersalivation, nausea, vomiting, hyperemesis, hematenesis, hypermotility, diarrhea, abdominal pain)
Second	Additional neuromuscular symptoms (advanced general paresthesia; paralysis of phalanges and extremities; pupillary dilatation, reflex changes)
Third	Increased neuromuscular symptoms (dysarthria; dysphagia, aphagia; lethargy; incoordination, ataxia; floating sensation; cranial nerve palsies; muscular fasciculations); cardiovascular/pulmonary symptoms (hypotension or hypertension; vasomotor blockade; cardiac arrhythmias including sinus bradycardia, asystole, tachycardia, and atrioventricular node conduction abnormalities; cyanosis; pallor; dyspnea); dermatologic symptoms (exfoliative dermatitis, betechiae, blistering)
Fourth	Respiratory failure, impaired mental faculties, extreme hypotension, seizures, loss of deep tendon and spinal reflexes

**Table 2:** Distribution of TTX in animals other than pufferfish.

Animals			Toxic parts	Maximal toxicity
Platyhelminthes	Turbellaria, Flatworms	*Planocera spp.*	Whole body	>1000 MU/g
Nemertinea	Ribbonworms	*Lineus fuscoviridis* *Tubulanus punctatus* *Cephalothrix linearis*	Whole body Whole body Whole body	>1000 MU/g 100–1000 MU/g >1000 MU/g
Mollusca	Gastropoda	*Charonia sauliae * *Charonia lampas lampas* *Babylonia japonica* *Tutufa lissostoma * *Zeuxis siquijorensis * *Niotha clathrata * *Niotha lineata * *Cymatium echo* *Pugilina ternotoma * *Pleurobranchaea maculata*	Digestive gland Digestive gland Digestive gland Digestive gland Whole body Whole body Whole body Digestive gland Digestive gland skin	>1000 MU/g 10–100 MU/g 100–1000 MU/g >1000 MU/g >1000 MU/g >1000 MU/g 10–100 MU/g 10–100 MU/g
	Cephalopoda	*Hapalochlaena maculosa*	Posterior salivary gland (adult), Whole body (semi-adult)	>1000 MU/g
Annelida	Polychaeta	*Pseudopolamilla occelata*	Whole body	10–100 MU/g
Arthropoda	Xanthidae crabs	*Atergatis floridus* *Zosimus aeneus*	Whole body Whole body	10–100 MU/g 10–100 MU/g
	Horseshoe crab	*Carcinoscorpius rotundicauda*	Egg	10–100 MU/g
Chaetognatha	Arrowworms	*Parasagitta spp. * *Flaccisagitta spp.*	Head Head	detected detected
Echinodermata	Starfish	*Astropecten spp.*	Whole body	100–1000 MU/g
Vertebrata	Pisces, Goby, Amphibia	*Yongeichthys criniger*	Skin, viscera, gonad	100–1000 MU/g
		*Tarica spp. *	Skin, egg, ovary, muscle, blood	100–1000 MU/g
		*Notophthalmus spp.*	Skin, egg, ovary	10–100 MU/g
	Newts	*Cynopsis spp. *	Skin, egg, ovary, muscle, blood	10–100 MU/g
		*Triturus spp.*	Skin, egg, ovary, muscle, blood	detected
	Frogs	*Atelopus spp.* *Colostethus sp.* *Polypedates sp. * *Brachycephalus spp.*	Skin Skin Skin Skin, liver	>1000 MU/g 100–1000 MU/g 100–1000 MU/g 100–1000 MU/g

**Table 3:** Pufferfish poisoning incidents in Japan.

Year	Number of incidents	Number of patients	Number of deaths	Mortality (%)
1965	106	152	88	57.9
1970	46	73	33	45.2
1975	52	75	30	40.0
1980	46	90	15	16.7
1985	30	41	9	22.0
1990	33	55	1	1.8
1995	30	42	2	4.8
1996	21	34	3	8.8
1997	28	44	6	13.6
1998	27	39	4	10.3
1999	20	34	2	5.9
2000	29	40	0	0.0
2001	31	52	3	5.8
2002	37	56	6	10.7
2003	28	35	3	8.6
2004	44	61	2	3.3
2005	40	49	2	4.1
2006	26	33	1	3.0
2007	29	44	3	6.8
2008	40	56	3	5.4
2009	24	50	0	0.0
2010	23	29	0	0.0

**Table 4:** TTX-bearing gastropods and food poisoning cases due to them in Japan.

Name of gastropod	Poisoning Year	Place	Number of patient	Number of death	Predatory habit
Ivory shell, *Babylonia japonica *	1957, Jun.	Niigata	5	3	Necrophagous
Trumpet shell,* Charonia sauliae *	1979, Dec.	Shizuoka	1	0	Carnivorous
	1982, Dec.	Wakayama	1	0	Carnivorous
	1987, Jan.	Miyazaki	2	0	Carnivorous
“Kinshibai,” *Alectricon glans *	2007, Jul.	Nagasaki	1	0	Necrophagous
	2008, Jul.	Kumamoto	1	0	Necrophagous

Frog shell, *Tutufa lissostoma *		Shizuoka			Carnivorous
“Hanamushirogai,” *Zeuxis siquijorensis *		Shizuoka			Necrophagous
“Araregai,” *Niotha clathrata *		Shizuoka			Necrophagous

Total			11	3	

**Table 5:** TTX-bearing gastropods and food poisoning cases due to them in other countries.

Taiwan and China					
Name of gastropod	Poisoning Year	Place	Number of patient	Number of death	Predatory habit

*Zeuxis samiplicutus*	1977–2001, Jun.	Zhoushan, China	310	16	Necrophagous
*Niotha clathrata*, *Zeuxis scalaris *	1994, May	Pingtung, Taiwan	26	0	Necrophagous
*Z. sufflatus*, *N. clathrata *	2001, Apr.	Taipei, Taiwan	5	0	Necrophagous
*Z. siquijorensis*	2002	Fujian, China	>20	>3	Necrophagous
	2004	China	55	1	
*Zeuxis *sp. and/or* Niotha *sp.	2002, Jul.	Tsingtao, China	3	0	Necrophagous
	2002-up to date	Fujian to Tsingtao, China	—	—	Necrophagous

Total			>419	>20	

New Zealand					

Sea slug, *Pleurobranchaea maculata *	2009, Jul.	Auckland		14 (dogs)	Herbivorous Carnivorous

**Table 6:** Primary TTX producers.

Marine bacteria	Source
*Vibrio alginolyticus*	From starfish
*Vibrio VIII*	From crab
*Shewanella algae*	From *Jania* sp.
*Alteromonas tetraodonis*	
*S. putrefaciens*	From pufferfish
Other marine bacteria	

**Table 7:** Toxicity of cultured pufferfish liver (1982–2009).

Culture	Year of collection	Age	Number of collection	Toxicity (MU/g)
Sea	1981–2003	1–3	4258	<2–<10
Land			4504	<2–<8
open system	2001–2009	1-2	4173	
closed system	2008-	1-2	331	

Total			8762	
